# Development and Application of Endoscopic Antireflux Mucosectomy in Treating Refractory Gastroesophageal Reflux Disease

**DOI:** 10.1155/grp/9564312

**Published:** 2025-09-11

**Authors:** Xiaoyu Hu, Jie Feng, Huimin Ma, Xiaojun Huang

**Affiliations:** Department of Gastroenterology, Lanzhou University Second Hospital, Lanzhou, Gansu Province, China

**Keywords:** antireflux mucosectomy, endoscopic mucosal resection, endoscopic therapy techniques

## Abstract

Gastroesophageal reflux disease (GERD) is primarily managed with acid suppressors, while laparoscopic fundoplication is considered the gold-standard surgical treatment for patients who have a suboptimal response to medical therapy, despite its limited acceptance. However, there have been alternative endoscopic treatment techniques available, including radiofrequency therapy, transoral fundoplication, and mucosal resection or mucosal ablation for this subgroup of patients, among which antireflux mucosectomy (ARMS) stands out as a relatively novel and minimally invasive option. The objective of this article is to provide gastroenterologists with a more comprehensive understanding of the technical features, current application status, clinical outcomes, and future perspectives regarding ARMS in the management of GERD. It is expected that ARMS will have a place in the standard endoscopic treatment of GERD. In the meantime, long-term multicenter, large-sample studies are required to provide a more convincing evaluation.

## 1. Introduction

Gastroesophageal reflux disease (GERD) is a chronic condition that contains intricate connections between the physiological and psychological aspects. Its prevalence continues to escalate with societal advancements. The 2019 Global Burden of Disease Report shows that countries such as Latin America have the highest age-standardized prevalence rate (ASPR) of GERD (12,000 cases per 100,000 population), and East Asia has the lowest ASPR, which is less than 5% [1]. A recent survey indicated that symptom-defined GERD was present in 5.6% (95% CI: 5.4%–5.8%) of Chinese adults [2]. The development of GERD is likely to be multifactorial, stemming from factors such as increased intra-abdominal pressure due to obesity, anatomical disorders like hiatal hernia of the esophagus, and transient lower esophageal sphincter relaxation (TLESR) [3]. Prolonged GERD can give rise to esophagitis, esophageal ulcers, esophageal strictures, Barrett's esophagus (BE), and esophageal adenocarcinoma [4].

Proton pump inhibitors (PPIs) and potassium-competitive acid blockers (P-CABs) are first-line drugs for the treatment of GERD [5]. Due to factors such as reflux hypersensitivity (RH) and nocturnal acid breakthrough (NAB), the efficacy is limited in approximately 40% of patients [6], with recurrence of reflux symptoms observed in about 75%–90% of patients upon discontinuation of the medication [7]. The term “refractory GERD” is described when symptoms have responded not completely or partially to a standard dose of PPI therapy after a sufficient period of therapy [8]. PPIs alone do not address anatomical defects and neuromuscular deficits, while long-term application of PPIs may lead to multiple side effects [9]. Laparoscopic fundoplication is the current standard antireflux surgery. Despite the instant efficacy of laparoscopic fundoplication in resolving recurrent symptoms, its utilization remains limited to less than 5% of patients due to concerns over adverse effects [10]. Laparoscopic fundoplication surgery can result in both acute and long-term complications. Approximately 50% of patients experience acute dysphagia, 10% of patients develop strictures after fundoplication, 10%-32% of patients have the syndrome of gastroparesis, and 18%–33% of patients experience diarrhea [11]. Approximately 25%–30% of GERD patients exhibit inadequate response to medication or experience long-term medication intolerance, yet decline surgical intervention [12]. Therefore, there is a necessity for novel and well-accepted therapeutic alternative options for GERD. There are currently four devices available for endoscopic treatment of GERD: a transoral incisionless fundoplication (TIF) device (EsophyX, EndoGastric Solutions, Redmond, WA), a radiofrequency energy delivery system (Stretta, Mederi Therapeutics Inc., Greenwich, CT), the Ultrasonic Surgical Endostapler device (MUSE, Medigus, Omer, Israel), and GERDx (G-SURG GmbH, Seeon-Seebruck, Germany), a recently launched endoscopic plication device. The first short-term outcomes of GERDx suggest an improvement in objective reflux parameters and quality of life [13, 14].

Apart from that, a range of novel endoscopic techniques for GERD have gradually emerged as alternative therapies, including endoscopic radiofrequency ablation (Stretta) [15], endoscopic full-thickness plication [16], filler injection, and implantation therapy [17]. The emergence of these endoscopic techniques addresses the unmet needs of patients who are “PPIs nonresponse but surgery-unwilling,” thereby bridging a crucial gap in their treatment. Wong et al. reported a retrospective study between the Nissen procedure and ARMS in patients with hiatal hernia, revealing that ARMS exhibited significant superiority over the Nissen procedure in terms of operative time, blood loss, and length of hospital stay [18]. It should be noted that in this study, patients with hiatal hernia larger than 2 cm were not included. Moreover, among the patients receiving ARMS treatment, approximately 30% eventually required surgical fundoplication. Therefore, further investigation of these endoscopic techniques is necessary to ascertain the long-term outcomes and evaluate the suitability of additional dedicated equipment and the implications of foreign body implantation to fully establish the value of its application. In contrast, ARMS is an endoscopic mucosal resection (EMR) or endoscopic submucosal dissection (ESD)–based technique that does not need specialized equipment. The semicircumferential EMR at the gastroesophageal junction (GEJ), where the resected area undergoes shrinkage during the healing process, represents a relatively safer and more effective approach. Based on the same principle, recent studies have also suggested using the solidification current or argon plasma coagulation (APC) to ablate the mucosa to induce scar formation, achieving similar results. This method is called antireflux mucosal ablation (ARMA). However, the postoperative bleeding rate was 4% (95% CI [0.01, 0.07], *I*^2^ = 19%) in patients who underwent ARMA and 2% (95% CI [0.00001, 0.04], *I*^2^ = 0%) in patients who underwent ARMS. The postoperative dysphagia rate was 17% (95% CI [0.09, 0.25], *I*^2^ = 42%) in patients who underwent ARMA and 9% (95% CI [0.01, 0.17], *I*^2^ = 59%) in patients who underwent ARMS. Finally, the sample size of patients receiving ARMA treatment is smaller than that of those receiving ARMS treatment [19]. Therefore, we have focused more on ARMS.

## 2. The Procedure of ARMS

### 2.1. Preassessment

It is important to evaluate the therapeutic effect using both subjective and objective tools. According to many studies, reflux symptoms significantly improved after endoscopic treatment (ARMS) [20]. There are many questionnaires related to symptoms, including the Gastroesophageal Reflux Disease Health-Related Quality of Life (GERD-HRQL), Gastroesophageal Reflux Disease Questionnaire (GERD-Q), Gastroesophageal Reflux Disease Symptom Frequency Scale (FSSG), SF-12 score [21], and Reflux Severity Index (RSI) [22]. Among them, GERD-HRQL is the most commonly used questionnaire. However, symptom questionnaires are subjective indicators and are greatly influenced by the patients. Twenty-four-hour esophageal pH monitoring, Demarest score, esophageal acid exposure time (AET), and Hill grade valve are the main objective data for evaluating the antireflux effect, and the assessment results are more reliable.

The inclusion criteria for the patients are as follows: (i) Clinically: PPI/P-CAB treatment was ineffective, or there was a response to PPI/P-CAB treatment but the patient was unwilling to take it for a long term and refused surgical treatment; (ii) 24-h reflux impedance–acidity monitoring: positive symptom index (SI) or symptom association probability (SAP); and (iii) high-resolution manometry (HRM) did not reveal any obvious primary esophageal motility disorder [23]. It should be noted that instances exist where PPIs exhibit partial efficacy in managing symptoms of GERD, leading to suboptimal PPI therapy [24, 25]. This requires drug withdrawal to meet the requirements of preoperative dynamic reflux monitoring and assessment. Further endoscopic procedures should be performed only when reflux monitoring confirms the presence of pathological acid reflux under insufficient PPI control.

### 2.2. ARMS Operational Procedures

ARMS was initially developed in a patient with BE-related lesions treated using ESD [26]. Although the specific surgical techniques of ARMS vary among studies, they are all based on the principle of scar tightening at the cardia following the destruction of the cardia mucosa [26].

### 2.3. Standard Procedure

**Figure 1:**
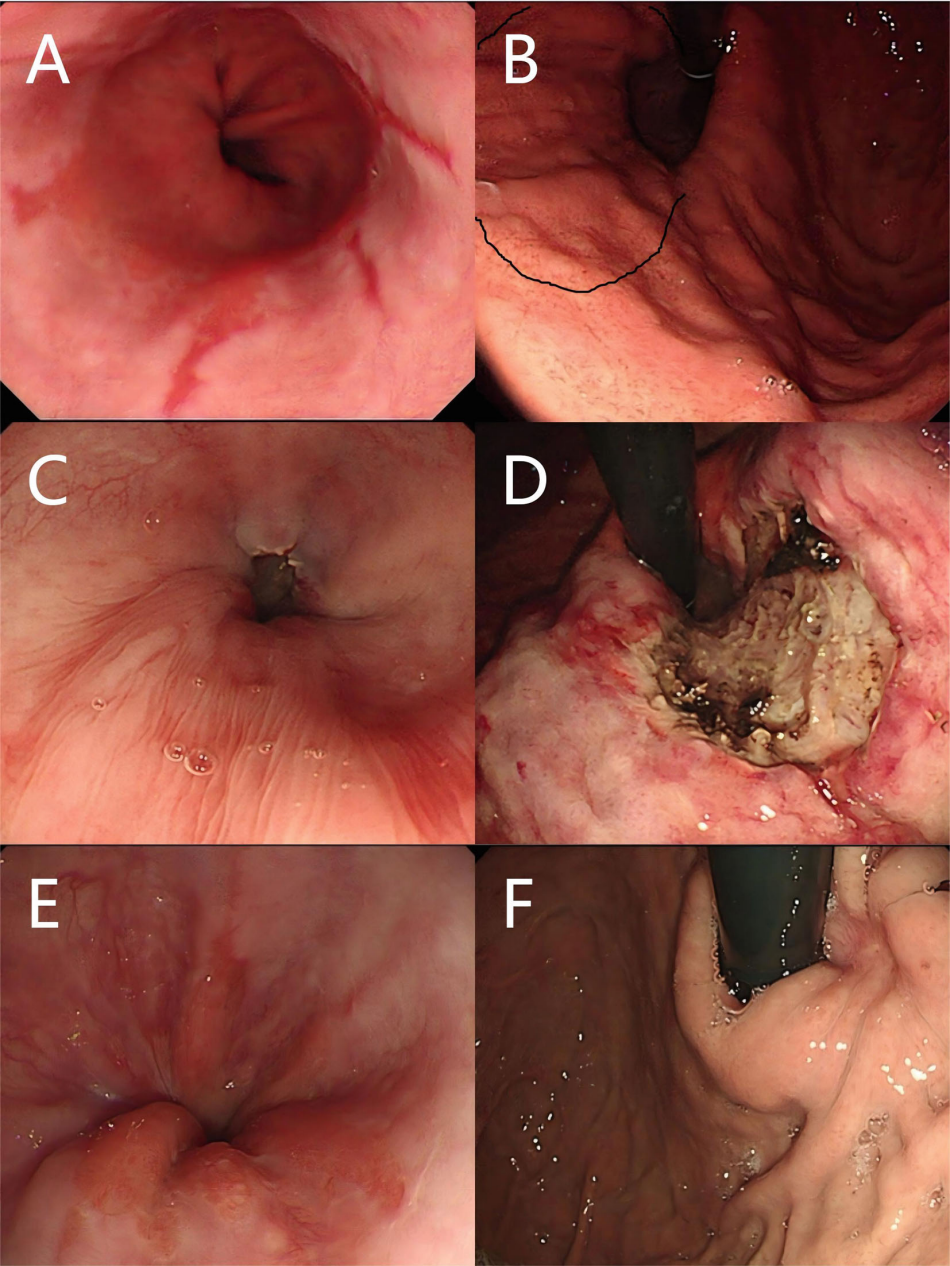
Standard procedure for ARMS. (A) Reflux esophagitis (LA-B). (B) The cardia is relaxed, and preresection range is marked. (C, D) Submucosal injection along the base of the cardia, then remove two-thirds of the circumferential mucosa using an endoloop. (E) Esophageal mucosa was normal after operation. (F) Usually after 2–6 months, the cardia is tight.

Standardized EMR/ESD techniques are employed for mucosal resection (Figure 1). Markings are made on the mucosa along the anticipated margins of resection. Mucosal resection is carried out along the lesser curvature of the cardia during semicircular ARMS. The length of mucosal resection at the cardia is measured by lateral retroflection of the stomach and ensured to be at least 3 cm in length (1 cm for the esophagus and 2 cm for the stomach). The retained length of mucosa on the greater curvature side is approximately twice the diameter of the endoscope in its retroflexed position, enabling it to form a well-defined, firm-textured mucosal flap in subsequent procedures [26].

### 2.4. Modified Procedure

**Figure 2:**
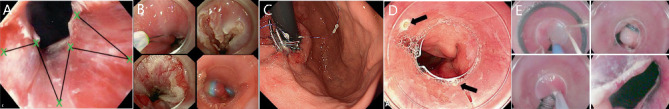
Schematic of different modified procedures. (A) ARMS-RAP. (B) L-ARMS. (C) ARM-P. (D) ARMS-C. (E) ARM-b.

Benias et al. [27] attempted to use a novel resection and plication (RAP) approach including a semiperipheral mucosal resection as well as full folding of the LES and cardia to recreate the angle of His, the length of the LES channel, the mucosal flap, and the high-pressure zone in the left posterolateral quadrant (Figure 2A). The ligation-assisted ARMS (L-ARMS) proposed by He et al. [28] utilized the inherent mucosal folds on the lesser curvature of the cardia, facilitating direct resection with a snare (Figure 2B). It only requires EMR without submucosal injection. Due to the strong contraction of the intrinsic muscular layer of the cardia, it is challenging for the intrinsic basal layer to be snared together with the mucosal layer, resulting in a low risk of perforation and minimal occurrence of serious complications such as hematemesis and esophageal perforation during or after surgery. These findings suggest that L-ARMS can be safely implemented in primary healthcare facilities and easily promoted. The conventional ARMS procedure requires a significant amount of time for scar tissue growth, resulting in no short-term benefits. In contrast, the L-ARMS procedure promotes rapid healing by inducing the narrowing of the mucosal incision through ligation of the mucosa around the squamous-column junction. Additionally, necrosis in the ligated area leads to ulcer formation, further enhancing cardia surface healing. Antireflux mucoplasty (ARM-P) (Figure 2C), incorporating the additional step of employing sutures for wound closure after mucosa removal, represents another enhanced alternative for facilitating wound healing. The study conducted by Yoo et al. [29] introduced a simplified cap-assisted ARMS (ARMS-C) procedure, which involves straightforward steps including injection, insertion of the loop through the hole, and subsequent excision of the lesion (Figure 2D). Considering the elimination of retroflexed positioning, avoidance of repeated endoscopic placements, and device removals, resulting in reduced operative times, it can be argued that the ARMS-C technique surpasses the L-ARMS technique. Hedberg et al. initially employed the banded ARMS approach (ARM-b) (Figure 2E), wherein a water cushion was injected into the submucosa to facilitate ligation of the targeted mucosa, followed by the use of a snare, and the banded tissue was transected using forced coagulation (effect 2, max W 40).

### 2.5. Derivative Procedure

Professor LingHu first reported peroral endoscopic cardial constriction (PECC) [30], in which the mucosal layer is ligated using a lancing device on the greater curvature and lesser curvature sides 1 cm above the dentate line, allowing natural scar formation. Technically, the PECC method is probably the simpler and less time-consuming procedure. However, the main factor influencing the efficacy was the depth of ligation. Inoue et al. [31] introduced ARMA, a technique involving the injection of indigo carmine into the submucosal layer after delta-knife electrocoagulation labeling, followed by ablating the mucosal layer using the delta-knife spray coagulation pattern. It would be a major challenge for patients who have received previous ESD for unrelated reasons and present with symptoms of refractory GERD. ARMA has a greater advantage over ARMS due to its repeatability. Although ARMA can tighten the GEJ, ARMS remains the best option for endoscopic treatment of GERD, considering that the removal of the cardia mucosa by ARMS may largely block the mechanism of transient relaxation of the LES. ARMA does not require tissue removal and carries a minimal risk of nerve damage. Compared to ARMS, ARMA offers precise control over the extent and depth of ablation, with an apparent low incidence of puncture-related complications. To achieve a comparable effect to ARMS, the ablation must be deep enough to access the submucosal space [31, 32].

## 3. Extent and Pattern of Excision

The size, location, and shape of the mucosal resection in ARMS are relatively variable, and there is no uniform standard at present. In the majority of ARMS studies, excision of the mucosa on the lesser curvature of the EGJ has been favored as it remodels the morphology of the gastroesophageal valve flap, thereby preserving elasticity on the greater curvature side (His angle side). Furthermore, the incidence of requiring additional antireflux surgery for the ARMS performed on the EGJ lesser curve is lower [18]. However, certain individual studies have proposed that excision of the greater curvature side is more advantageous due to its scar formation potential and ability to enhance sling fiber function [27]. However, this issue is subject to further controlled studies.

In the study presented by Inoue et al. [26], the initial two patients underwent circumferential resection for BE with high-grade intraepithelial neoplasia, and both developed postoperative strictures despite excellent antireflux results. Subsequently, a crescent resection technique was implemented [33], demonstrating comparable efficacy in managing reflux. Approximately 2/3~4/5 of the circumferential mucosa was resected centered on the cardiac mucosa overall. To date, limited research has been conducted on the impact of varying resection ranges on both the efficacy and adverse effects of ARMS. A comparative study examining resection ranges of 270° versus 180° suggests that 180° ARMS may have the advantage of fewer new dysphagia complaints compared with 270° ARMS and may be more appropriate for the treatment of refractory GERD [34]. However, there was no statistically significant difference observed between 180° ARMS and 270° ARMS in terms of reflux control, relief of GERD symptoms, improvement in quality of life, and objective indicators of GERD. Another study [35] that reached similar conclusions demonstrated no significant difference in the improvement of GERD-Q, DeMeester score, and AET index between the 3/4 and 2/3 circumferential submucosal resection groups at a postoperative follow-up of 6 months. However, the incidence of postoperative esophageal stricture was higher in the 3/4 resection group than in the 2/3 resection group.

The length and pattern of the ARMS may also affect the outcome of the surgery. A study suggested that mucosal resection should be performed at a distance of 1 cm from the esophagus and 2 cm from the stomach [26]. Of which, the submucosal resection from the stomach side may have a greater effect against reflux. Additionally, compared to crescent resection, butterfly-shaped resection demonstrates superior efficacy in reducing dysphagia risk [36]. Among the 21 patients who underwent ARMS using the butterfly method, only one required balloon dilation, while 12 out of 81 patients who underwent crescent resection experienced stricture.

Further research is needed to strike a balance between effective reflux control and the prevention of dysphagia. Additionally, the extent of mucosal resection should be determined based on the degree of relaxation of the cardia and esophageal contractile function. It is common to experience a temporary narrowing of the esophagus 2–3 weeks after surgery, so when dealing with individuals with poor esophageal motility, the extent of mucosal resection needs to be reduced compared to the usual case. The purpose of this approach is to avoid resection of the squamous mucosa of the esophagus, which can reduce the rate of stricture [18]. Simultaneously, it aims to preserve a wider area on the greater curvature to maintain the structural integrity of the His angle [37].

### 3.1. Clinical Efficacy

#### 3.1.1. Technique Evaluation of ARMS

As depicted in Table 1, despite the derivation of multiple modified versions from it, they do not compromise the success rate or escalate the need for additional procedures. The duration of the procedure is influenced by operator experience, individual patient variations, and technique characteristics. Thus, there is considerable heterogeneity in the results of studies. Postoperative clinical outcomes demonstrate an effective rate of approximately 70% or higher and a reoperation requirement probability below 15%. Noncomparative [38, 39] studies and RCT [40] on TIF have shown clinical success rates ranging from 50% at 12 months to 92% at 10 years. There is no absolute advantage over ARMS. Even in cases where ARMS fails, patients can still undergo subsequent antireflux surgery, indicating that ARMS failure does not impede further treatment. Regarding the duration of postoperative follow-up, a majority of studies have reported a period of 6 months. Standardization in this regard is yet lacking. The length of follow-up should be determined based on the severity of patient symptoms, surgical intervention, and postoperative outcomes.

## 4. Clinical Responses From Follow-Up Investigation

### 4.1. Evaluation of Clinical Symptom Improvement

As listed in Table 2, most studies used improvement in clinical symptoms as the primary criterion for assessing effectiveness. The current studies have all reported significant improvement in GERD symptoms in patients after ARMS. Symptom scores used in different studies are not identical. However, only 66% of the patients who underwent TIF treatment demonstrated clinical improvement [41] (with clinical improvement defined as a GERD-HRQL score improvement of at least 50% or relief of heartburn and regurgitation). The most frequently used assessment criteria are the GERD-Q, the GERD-HRQL score, and the FSSG. Because of its subjective nature and varying criteria employed, comparing results across different studies is challenging. The questionnaire scores are also influenced by the placebo effect of undergoing treatment. Nonetheless, existing data provide compelling evidence supporting ARMS' effectiveness.

The discontinuation of PPIs is another component that reflects the improvement of clinical symptoms. During the postoperative follow-up of ARMS, a significant proportion of patients (ranging from 42.0%–89.6%) was able to completely discontinue PPIs [18, 36], thus confirming the superior efficacy of ARMS in PPI-refractory GERD treatment. The discontinuation rate of PPIs following TIF was only 4.2% [41]. Moreover, this finding also highlights the effectiveness of ARMS in potentially preventing PPI overuse.

### 4.2. 24-h Esophageal Dynamic pH Monitoring

The DeMeester score and AET are both robust objective indicators for evaluating acid reflux, as demonstrated by the significant improvement in both measures at postoperative follow-up compared to preoperative values, as shown in Table 2 [26, 28, 30, 34, 36, 42]. Compared to subjective scoring systems, the results of the DeMeester score and AET are more reliable. However, the poor comfort associated with 24-h esophageal dynamic pH monitoring via nasal cannula leads to low adherence to follow-up. Particularly for patients experiencing symptom relief, a majority of them are unwilling to cooperate with this test during the follow-up period, resulting in inadequate evaluation of objective indicators regarding postoperative relief in GERD patients after ARMS. Consequently, only nearly one-fourth of the preoperative and postoperative pH monitoring data can be obtained before and after ARMS [36]. However, the average suppression of AET by ARMS was superior to that of Stretta, as several cohort studies [43] and RCT [44] assessing the Stretta procedure reported no statistically significant alteration in AET 6 months postprocedure.

### 4.3. Evaluation of Gastroesophageal Flap Valve (GEFV) Morphology

**Figure 3:**
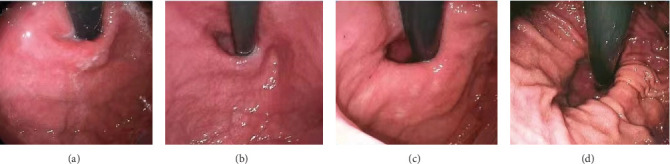
Hill classification of gastroesophageal valve. (a) Grade I: The edge of the tissue ridge is obvious, tightly wrapping the endoscope along the lesser curvature. (b) Grade II: The ridge is not as obvious as Grade I, but it occasionally opens with respiration and closes promptly. (c) Grade III: The ridge is barely present and cannot tightly wrap the endoscope. (d) Grade IV: There is no ridge at all. The gastroesophageal area is open, and the esophageal squamous epithelium is readily visible.

Previous studies have demonstrated the association between GEFV morphology and symptoms of GERD [45]. According to the standards set by Hill et al., GEFV is divided into four levels [46]. The sample images of the Hill classification standard are shown in (Figure 3). Therefore, the observed reduction in Hill score during postoperative review provides morphological evidence supporting the effectiveness of ARMS. As depicted in Table 2, several studies have reported significant improvement or restoration of Hill score to 1 following surgery, visually confirming the remarkable efficacy of ARMS.

## 5. Indications and Contraindications

The indications are summarized as follows: patients with a definite diagnosis of GERD who have been effectively treated with acid-reducing medication. However, they are reluctant to take long-term or cannot tolerate the side effects of the medication [47]. Contraindications to ARMS include [33, 36, 42, 48] (1) various special conditions, such as pregnancy, lactation, abnormal obesity, severe mental illness, and substance abuse; (2) esophageal hiatal hernia > 2 cm in length, presence of achalasia or GEJ outflow tract obstruction (according to the Chicago Classification, Version 4.0), high-grade esophagitis (LA Class C or D), BE, eosinophilic esophagitis, and other organic esophageal diseases; and (3) other contraindications to endoscopic procedures should be considered. Relative contraindications may arise from a combination of other organ and system diseases including heart failure, end-stage renal disease, cirrhosis, malignant tumors, a history of previous esophageal/gastric surgeries, and bleeding tendency. However, the decision regarding the treatment plan will be made after careful consideration of the risks and benefits.

## 6. Complications

The complication rate of ARMS was 17.2% (95% CI, 13.1~22.2). Dysphagia/esophageal stricture was the most common adverse effect, with an incidence of 11.4%, higher than surgical fundoplication [49]. It was followed by hemorrhage at rates of approximately 5.0%. A study by Hedberg et al. suggested that discontinuation of PPIs immediately after ARMS may contribute to a higher occurrence of postoperative dysphagia, as gastric acid stimulation can potentially accelerate scar formation. Therefore, it is recommended to continue PPI therapy for 2–4 weeks after ARMS to minimize the inflammatory response and reduce scarring intensity, thereby decreasing the likelihood of postoperative strictures and dysphagia. Based on previous experience, the risk of esophageal stricture is 80%–100% when more than three-fourths of the peripheral esophageal mucosa is removed [50]. Even in cases with esophageal stricture, most patients still achieve satisfactory swallowing function and experience successful antireflux outcomes following dilation therapy. With advancements in surgical techniques, complications such as bleeding and perforation can largely be avoided, thus posing no significant threat to the safety of ARMS.

## 7. Current and Future Perspectives

In the past three decades, with advancements in endoscopic technology, an increasing number of endoscopic treatments for GERD have been proposed and implemented in clinical practice. Depending on the different repair schemes for the antireflux barrier, endoscopic antireflux surgery can be divided into four categories: mucosal injection–type (swelling agent: bovine collagen protein), radiofrequency-type (Stretta), LES reconstruction–type (TIF) [14], and scar repair–type. The first type is not considered due to a short duration of maintenance and the need for repeated treatments, which pose a significant risk of complications. The second and third types are not often adopted because their operation procedures are rather complicated and the costs are high [51]. Among them, ARMS has garnered increasing attention owing to its safety and simplicity, including narrow ARMS and various modified ARMS procedures. ARMS has been approved for reimbursement in Japan in April 2022, and it will be a convenient and effective treatment option for patients with refractory GERD.

Currently, the research population for ARMS is limited and heterogeneous, posing challenges to generalizability. The inclusion criteria for refractory GERD patients in the studies lack consistency, while the excluded population comprises individuals who are more likely to exhibit refractory GERD symptoms. It is important to mention that definitions of refractory GERD have varied significantly across studies, likely due to the absence of standardized diagnostic criteria. This heterogeneity contributes to the inconsistent outcomes observed with both endoscopic and surgical therapies. The scope of ARMS surgery extends beyond patients with refractory GERD, encompassing those with laryngopharyngeal reflux disease (LPRD) who are PPI dependent or unable to tolerate the adverse effects of PPIs, thereby offering a novel and efficacious treatment option [52]. The Rome IV definition of RH includes retrosternal symptoms (such as heartburn or chest pain), normal endoscopy results, and normal AET but positive SI or SAP [33, 53]. Symptomatic improvement was also obtained in RH patients treated with ARMS [33]. This suggests that ARMS may represent a promising therapeutic approach for managing cases of RH. The folds induced by NF and TIF tend to loosen over time. ARMS is a safe, effective, and clinically feasible option for patients with a history of NF or TIF surgeries [54].

Although ARMS offers an opportunity to treat patients with refractory GERD, the current level of evidence from studies related to ARMS is insufficiently adequate. Firstly, the current sample size of studies is small and should be further enlarged for improved reliability. Secondly, the duration of follow-up is relatively short, as indicated in Table 1, limited to 3 years for most patients. While short-term efficacy has been sufficiently demonstrated for ARMS, there remains uncertainty regarding its long-term effectiveness compared to surgical procedures that more comprehensively reconstruct the antireflux barrier. Compared with other endoscopic antireflux surgeries, both ARMS and ARMA have demonstrated good efficacy in improving symptoms related to GERD [19]. Lastly, controversy surrounds the mechanism of action for ARMS. Two hypotheses exist. One suggests that it primarily enhances the antireflux barrier while another proposes damage to esophageal nerves resulting in symptom reduction. Objective examinations have also revealed that ARMS increases overall relaxation pressure and LES resting pressure while decreasing GEJ distensibility, which may reduce TLESR frequency [55]. Simultaneously, there are also some cases where the results of pH-impedance monitoring before and 24 h after ARMS show that the gastroesophageal reflux frequency is almost not improved, although the GERD symptoms are significantly improved. It cannot be excluded that the reason may be that during mucosal dissection, high-frequency waves cause partial destruction of sensory nerve endings in the smooth muscle layer of GEJ and weaken RH similar to Stretta. It is also possible that fibrosis following ARMS prevents the delivery of chemicals to sensory nerve endings. Therefore, although objectively reducing reflux occurrence seems plausible with ARMS usage, whether it affects associated nerves still requires clarification.

## 8. Conclusion

In summary, ARMS is beneficial for a carefully selected subset of GERD patients. This method has gained recognition as a promising standardized endoscopic treatment for GERD in clinical practice owing to its favorable efficacy, safety profile, and procedural simplicity. But surgical interventions remain the standard of care in patients with large hiatal hernias, Hill's Grade III or IV valves, and BE.

## Figures and Tables

**Table 1:** Technology evaluation of ARMS in clinical application.

**Patients ( ** **n** ** )**	**ARMS types**	**Operative time**	**Technical success rate (%)**	**Clinical responses (%)**	**Additional procedures rate (%)**	**Follow-up time**	**References**
10	ARMS	EMR: 42–124, 76 min ESD: 98–176, 127 min	100%	100%	0	2 months	Zhang et al. [1]
13	ARMS	67.7 min	100%	92%	0	6 months	Liu et al. [2]
47	PECC	6.5 (3–19) min	100%	73.7%	0	6 months	Tack and Pandolfino [3]
10	RAP	44 (27–60) min	100%	80%	0	5–27 months	Katzka and Kahrilas [4]
62	ARMS	N	100%	87.1%	0	2, 6, and 12 months	Gyawali and Fass [5]
19	ARM-b	40.7 (17–64) min	100%	68%	15.8% (3/19)	6 months	Naik et al. [6]
24	ARM-B	35 ± 11 min	100%	81%	4.2% (1/24)	5–15 months	El-Serag et al. [7]
33	ARMS-C	31.2 (15–68) min	100%	93.9%	6.1% (2/33)	6 months	Fock et al. [8]
6	ARMS	< 40 min	83.3%	50%	0	3 months	Malfertheiner et al. [9]
33	ARMS	36 ± 13 min	100%	90.9%	0	6 months, 1 year, and 2 years	Broeders et al. [10]
39	ARMS	180° ARMS: 35 (29–58) min 270° ARMS: 55.5 (43–70) min	100%	100%	0	6 months	Yadlapati et al. [11]
109	ARM-B	54.7 ± 27.0 min	100%	69.3%	12.8% (14/109)	2 months to 3 years	Kamolz and Pointner [12]
69	L-ARMS	33 min	100%	85.5%	2.9% (2/69)	6 months	Weitzendorfer et al. [13]
12	ARMS	79.2 ± 23.9 min	100%	75%	0	3 months to 2 years	Kalapala et al. [14]
11	ARMS	50 (46~56) min	100%	82.6%	0	3–12 months	Sowa and Samarasena [15]
20	ARM-P	75 (45–205) min	100%	70%	0	1–7 months	Chuttani et al. [16]
96	ARMS-C	35 (10–60)	100%	86%	0	6 months to 2 years	Kamler et al. [17]

**Table 2:** The efficacy of ARMS for refractory GERD.

**Patients ( ** **n** ** )**	**DeMeester**		**GERD-Q/HRQL**		**AET**		**Hill score**		**Adverse events (** **n** **)**	**PPIs/P-CAB**	**References**
	**Pre**	**Post**	**Pre**	**Post**	**Pre**	**Post**	**Pre**	**Post**			
10	5.1	0.8	*N*	*N*	29.1	3.1	3.2	1.2	Dysphagia: 2	Discontinued: 10	Zhang et al. [1]
13	*N*	*N*	*N*	*N*	10.4 ± 15.5	7.4 ± 10.6	*N*	*N*	Dysphagia: 1	Discontinued: 3/reduced: 3/the usual: 6	Liu et al. [2]
19	61.2 (45.7–149.4)	23.1 (8.9–46.7)	*N*	*N*	207.0 (147.5–524.0)	72.0 (24.5–167.3)	*N*	*N*	Dysphagia: 2/bleeding: 1	*N*	Tack and Pandolfino [3]
10	*N*	*N*	26.6 ± 3.9	4.3 ± 2.4	*N*	*N*	3	1	Dysphagia: 1	Discontinued: 6/reduced: 4	Katzka and Kahrilas [4]
62	76.8 (18.3)	14.3 (6.1)	10.6 (1.9)	3.4 (1.5)	*N*	*N*	*N*	*N*	Dysphagia: 3	*N*	Gyawali and Fass [5]
19	*N*	*N*	*N*	*N*	*N*	*N*	*N*	*N*	Dysphagia: 3/bleeding: 1	Discontinued: 13	Naik et al. [6]
33	14.3 (10.9)	7.7 (9.4)	11.1 (3.1)	6.8 (3.1)	3.1 (3.1)	1.8 (2.4)	3	1	Dysphagia: 2	Discontinued: 21/reduced: 10	Fock et al. [8]
6	*N*	*N*	13.3 (1.1)/30.6 (7.7)	6.2 (4.0)/6.8 (3.7)	*N*	*N*	3	1	Dysphagia: 1/bleeding: 1	Discontinued: 1/reduced: 2/the usual: 3	Malfertheiner et al. [9]
33	*N*	*N*	16 (12)	6 (7.1)	*N*	*N*	3	1	Dysphagia: 3/bleeding: 1	Discontinued: 90.9%	Broeders et al. [10]
109	64.4 ± 75.7	24.9 ± 36.0	9.4 ± 2.7	6.6 ± 2.5	20.8 ± 24.3	6.9 ± 10.4	*N*	*N*	Dysphagia: 13/bleeding: 2	Discontinued: 42%–51%	Kamolz and Pointner [12]
39	*N*	*N*	11.38 ± 40	6.60 ± 63	18.5 ± 7.5 19.4 ± 7.2	7.9 ± 4.6 6.8 ± 5.2	3 (2–4)	1.5 (1–3) 1 (1–3)	Dysphagia: 9/bleeding: 1	Discontinued: 58.97%	Yadlapati et al. [11]
69	27.3 ± 22.7	12.0 ± 7.1	*N*	*N*	9.5 (0.9–25.3)	2.1 (0.2–17.2)	3 (1–4)	1 (1–3)	0	Discontinued: 55.1%/reduced: 30.4%	Weitzendorfer et al. [13]
23	15.4 (9.65–21.32)	7.44 (3.87–16.75)	9 (6~10)	52%–70% cases↓	4.6 (2.8~6.9)	2.1 (1.1~5.6)	II: 4 (17.4) III: 19 (82.6)	74% cases↓	Bleeding: 1	Discontinued: 15	Sowa and Samarasena [15]
12	21.2 (17.0–55.1)	4.8 (1.1–31.8)	*N*	*N*	6.6 (5.2–15.7)	1.0 (0.2–9.8)	*N*	*N*	Dysphagia: 3	*N*	Kalapala et al. [14]
20	*N*	*N*	9 (2–15)	7 (1–11)	27.5 (0–338.9)	↓ (significantly)	3 (1–4)	1 (1–2)	0	*N*	Chuttani et al. [16]
96	4.75 (0–67.8)	3.9 (0–40)	10.67 ± 3.06	7.55 ± 2.77	2.81 ± 2.56	2.07 ± 3.10	2 (1–4)	1 (1–4)	Dysphagia: 9	Discontinued: 5/reduced: 14/the usual: 3	Kamler et al. [17]

^↓^Represents that the corresponding postoperative indicators have decreased compared to the preoperative ones.

## Data Availability

The data that support the findings of this study are available from the corresponding author upon reasonable request.
